# ﻿New species of *Tropicoporus* (Basidiomycota, Hymenochaetales, Hymenochaetaceae) from India, with a key to Afro-Asian Tropicoporus species

**DOI:** 10.3897/mycokeys.102.117067

**Published:** 2024-02-05

**Authors:** Sugantha Gunaseelan, Kezhocuyi Kezo, Samantha C. Karunarathna, Erfu Yang, Changlin Zhao, Abdallah M. Elgorban, Saowaluck Tibpromma, Malarvizhi Kaliyaperumal

**Affiliations:** 1 Centre for Advanced Studies in Botany, University of Madras, Guindy Campus, Chennai 600025, Tamil Nadu, India University of Madras Chennai India; 2 Center for Yunnan Plateau Biological Resources Protection and Utilization, College of Biological Resource and Food Engineering, Qujing Normal University, Qujing, Yunnan 655011, China Qujing Normal University Yunnan China; 3 National Institute of Fundamental Studies (NIFS), Kandy, Sri Lanka National Institute of Fundamental Studies (NIFS) Kandy Sri Lanka; 4 Department of Biology, Faculty of Science, Chiang Mai University, Chiang Mai 50200, Thailand Chiang Mai University Chiang Mai Thailand; 5 College of Biodiversity Conservation, Southwest Forestry University, Kunming 650224, China Southwest Forestry University Kunming China; 6 Department of Botany and Microbiology, College of Science, King Saud University, Riyadh 11451, Saudi Arabia King Saud University Riyadh Saudi Arabia

**Keywords:** DNA, *Inonotuslinteus* complex, mushroom, new species, taxonomy, wood decaying fungi

## Abstract

The *Inonotuslinteus* complex, predominantly reported from East Asia, Mesoamerica and Caribbean countries, was circumscribed into *Tropicoporus* as one of the new genera, based on morphological and phylogenetic data. The present paper describes four new species of *Tropicoporus* from India. Morphological characteristics and phylogenetic analyses, based on ITS and nLSU data, delimited the new species, which are named *T.cleistanthicola*, *T.indicus*, *T.pseudoindicus* and *T.tamilnaduensis*. The pairwise homoplasy index (PHI) test was done to confirm the distinctive nature of the new species. The traits of Indian species remain distinct from one another, except for the pileate basidiome with the mono-dimitic hyphal system, cystidioles and broadly ellipsoid basidiospores. Descriptions, illustrations, PHI test results and a phylogenetic tree to show the position of the new species are provided. In addition, an identification key to *Tropicoporus* in Asia and an African species is given.

## ﻿Introduction

The morpho-taxonomy and phylogenetic analyses, based on the nLSU and ITS genetic markers, revealed that the *Inonotuslinteus* complex comprises two clades and are respectively treated as two new genera, *Sanghuangporus* and *Tropicoporus* (Zhou et al. 2015). *Tropicoporus* is characterised by their annual to perennial, resupinate, effused-reflexed to pileate basidiome with glabrous, uncracked to radially cracked pilear surface, homogeneous to duplex context, with or without a black line. A mono-dimitic or dimitic hyphal system with simple septate generative hyphae, presence or absence of cystidioles, presence of hymenial setae with smooth, fairly thick-walled to thick-walled, yellowish, subglobose to ellipsoid basidiospores are microscopic characteristic features of *Tropicoporus* (Zhou et al. 2015; Wu et al. 2022).

A total of forty-eight *Tropicoporus* species have been recorded in MycoBank with fifteen new species and thirty-three new combinations (as of 12 January 2024). Two new species, namely *Tropicoporusexcentrodendri* L.W. Zhou & Y.C. Dai and *T.guanacastensis* L.W. Zhou, Y.C. Dai & Vlasák have been delimited, based on nLSU and ITS datasets (Zhou et al. 2015). In addition, *Tropicoporusboehmeriae* (L.W. Zhou & F. Wu) Y. C. Dai & F. Wu, *T.drechsleri* Salvador-Montoya & Popoff, *T.flabellatus* V.R.T. Oliveira, J.R.C. Oliveira-Filho, Xavier de Lima & Gibertoni, *T.nullisetus* Xavier de Lima, V.R.T. Oliveira & Gibertoni, *T.stratificans* Y.C. Dai & F. Wu. and *T.texanus* A.A. Brown, D.P. Lawr. & K. Baumgartner were reported across the world, based on the morphological and molecular data (Wu et al. 2015; Coelho et al. 2016; Salvador-Montoya et al. 2018; Brown et al. 2019; Lima et al. 2022). Recently, seven new species viz., *T.angustisulcatus* Y.C. Dai & F. Wu, *T.hainanicus* Y.C. Dai & F. Wu, *T.lineatus* Y.C. Dai & F. Wu, *T.minus* Y.C. Dai & F. Wu, *T.ravidus* Y.C. Dai & F. Wu, *T.substratificans* Y.C. Dai & F. Wu and *T.tenuis* Y.C. Dai & F. Wu with twenty-four new combinations were reported, based on the combined dataset of ITS and nLSU sequences (Wu et al. 2022). Of the forty-eight legitimate *Tropicoporus* species, only *T.nullisetus* was reported without setae (Lima et al. 2022).

*Tropicoporuslinteus* (also known as *Phellinuslinteus*) is used as a renowned Chinese medicine. Due to the presence of *P.linteus* polysaccharides (PLPs), it may play a vital role in anti-aging, anti-bacterial, anti-inflammation, anti-tumour, anti-oxidant, hepatoprotective and hypoglycaemic processes (Chen et al. 2019). On the other hand, *Tropicoporustropicalis* has been reported to cause diseases in humans (Sutton et al. 2005; Haidar et al. 2017; Gupta et al. 2022).

In India, hymenochaetoid fungi from Himachal Pradesh were studied (Kaur et al. 2022). Fourteen hymenochaetoid members were documented from Tamil Nadu (Natarajan and Kolandavelu 1998). Nevertheless, studies on Indian hymenochaetoid fungi, based on molecular data have not been attempted, which makes it difficult to understand their evolutionary history, phylogenetic relationships and accuracy of species delimitation. This study is the first attempt to describe new *Tropicoporus* species from India, based on morphology and molecular evidence. In addition, an identification key to Afro-Asian *Tropicoporus* is given.

## ﻿Materials and methods

### ﻿Morphological analyses

Eight specimens were collected from parts of Eastern Ghats and the plain region of Tamil Nadu, southern India. Macro-morphological characteristics such as shape, size of basidiome, perennial or annual, colour, texture, margin (acute or obtuse), context (homogenous, duplex with or without black line), tube layer (colour, length, stratification) and pores (size and shape) were examined in the fresh sample and recorded. Colour descriptions were based on the Methuen Handbook (Kornerup and Wanscher 1978). To analyse the micro-morphological characteristics, free-hand sections of dry specimens were mounted in water, 5% potassium hydroxide (KOH) (v/w), cotton blue (CB) and Melzer’s reagent (IK). Sections were studied and photos were taken at magnification up to 1000× using a LABOMED OPTIC-CX BINO LED microscope. The drawings were made using LABOMED CxL2 compound microscope. Microscopic measurements and illustrations were made in 5% KOH solution. Basidiospores measurements (as minimum-mean-maximum) and Q values (length/width ratios) were recorded. The following abbreviations are used: IKI ¯ (inamyloid), IKI^+^ (amyloid), CB ¯ (acyanophilous), CB^+^ (cyanophilous), L = mean spore length (arithmetic average of all spores), W = mean spore width (arithmetic average of all spores), Q = (variation in the L/W ratios; and basidium length excludes the lengths of the sterigmata) and n = number of spores measured. For measuring the spores, an average of 50 spores were considered. Specimens in this study were deposited in the Madras University Botany Laboratory (MUBL), Centre for Advanced Studies in Botany, University of Madras, India.

### ﻿Genomic DNA extraction, PCR amplification and sequencing

Extraction of total genomic DNA from mycelium and dried basidiome followed the protocol of Doyle and Doyle (1987), modified by Góes-Neto et al. (2005). The ITS and nLSU regions were amplified and sequenced with the primers ITS1/ITS4 and LR0R/LR7, respectively (Vilgalys and Hester 1990; White et al. 1990). The polymerase chain reaction (PCR) procedure for ITS was as follows: initial denaturation at 95 °C for 3 min, followed by 32 cycles at 95 °C for 30 s, 52 °C for 30 s and 72 °C for 1 min and a final extension of 72 °C for 3 min. The PCR procedure followed for nLSU was as follows: initial denaturation at 94 °C for 1 min, followed by 34 cycles at 94 °C for 30 s, 45 °C for 30 s and 72 °C for 1.5 min and final extension at 72 °C for 10 min. The PCR products were sequenced at Eurofins Genomics India Pvt. Ltd., Karnataka, India.

### ﻿Phylogenetic analyses

The dataset comprised ITS and nLSU sequences of *Fulvifomes*, *Inonotus*, *Phellinus*, *Phylloporia*, *Sanghuangporus* and *Tropicoporus* retrieved from GenBank (NCBI), along with the outgroup (*Fomitiporellacaryophylli*, CBS 448.76) and the newly-generated sequences (deposited at GenBank (Sayers et al. 2023); for accession numbers, see Table 1). The dataset was aligned using MEGA X v.10.0.2 configured for Windows and edited manually to increase the alignment similarity (Kumar et al. 2018). The Maximum Likelihood (ML) tree was constructed using raxmlGUI 2.0 (Edler et al. 2020) with the best-fit evolutionary model estimated by jModelTest 2.1.10 with 1000 rapid bootstrap inferences (BS) (Guindon and Gascuel 2003; Darriba et al. 2012). Bayesian Inference (BI) was performed using MrBayes 3.2.7a with two independent runs and six chains of Metropolis-coupled Markov Chain Monte Carlo iterations for 2,000,000 generations and trees were sampled every 100 generations (Ronquist et al. 2012). A proportion of 0. 25% of all trees (nLSU+ITS, ITS, nLSU) were discarded as burn-in. The final alignments and the retrieved topologies were deposited in TreeBASE (http://purl.org/phylo/treebase/phylows/study/TB2:S31000).

**Table 1.** Names, strain numbers, countries of collection and the corresponding GenBank accession numbers of the sequences used in this study.

Species	Strain numbers	Country	Accession numbers	
			ITS	nLSU
*Fomitiporellacaryophylli*	CBS 448.76	–	AY558611	AY558611
*Fulvifomescentroamericanus* ^T^	JV0611_III	Guatemala	KX960763	KX960764
*F.elaeodendri*	CMW47825	South Africa	MH599094	MH599134
*F.nilgheriensis*	CBS 209.36	USA	AY558633	AY059023
*F.thailandicus* ^T^	LWZ 2014073-11	Thailand	KR905672	KR905665
*Inonotuspachyphloeus*	Wu 0407.6	Taiwan	KP030785	KP030770
*Phellinuslaevigatus*	CBS 122.40	USA	MH856059	MH867554
*P.populicola* ^T^	CBS 638.75	Finland	MH860960	MH872729
*Phylloporianodostipitata*	FLOR:51153	Brazil	KJ639057	KJ631414
*Sanghuangporusalpinus*	Cui12485	China	MF772781	MF772799
*S.baumii*	Cui 11769	China	MF772784	MF772803
*S.lonicericola*	Dai 8376	China	JQ860308	MF772805
*S.lonicerinus*	Dai 17093	China	MF772788	MF772807
*S.quercicola*	Dai 13947	China	KY328309	MF772809
*S.sanghuang*	Cui 14419	China	MF772789	MF772810
*S.vaninii*	DMR 95-1-T	North America	KU139198	KU139258
*S.vitexicola*	Wu 2006-21	–	MT906620	MZ437416
*S.weigelae*	Dai 16077	China	MF772794	MF772815
*S.zonatus*	Dai 10841	China	JQ860306	KP030775
*Tropicoporusangustisulcatus*	Dai 17409	Brazil	MZ484584	MZ437417
*T.angustisulcatus* ^T^	JV 1808/83	French Guiana	MZ484585	MZ437418
*T.boehmeriae* ^T^	LWZ 20140729-10	Thailand	KT223640	MT319393
*T.boehmeriae*	LWZ 20140729-13	Thailand	KT223641	MT319394
	Dai 20522	China	MZ484586	MZ437419
	Dai 20617	China	MZ484587	MZ437420
***T.cleistanthicola* ^T^**	**MUBL1089**	**India**	**OR272292**	**OR272337**
***T.cleistanthicola***	**MUBL1090**	**India**	**OR272291**	**OR272336**
*T.cubensis*	MUCL 47113	Cuba	JQ860324	KP030777
	MUCL 47079	Cuba	JQ860325	KP030776
*T.dependens*	JV 0409/12-J	USA	KC778777	MF772818
*T.detonsus*	CBS 617.89	–	AF534077	AY059037
	IDR 1300012986	USA	KF695121	KF695122
*T.drechsleri* ^T^	CTES:570140	Argentina	MG242439	MG242444
*T.drechsleri*	CTES:570144	Argentina	MG242437	MG242442
*T.excentrodendri*	Yuan 6234	China	KP030791	–
	Yuan 6229	China	KP030789	–
*T.flabellatus* ^T^	VRTO873	Brazil	MT908376	MT906643
*T.guanacastensis*	O 19228	Costa Rica	KP030794	–
*T.guanacastensis* ^T^	JV 1408_25	Costa Rica	KP030793	KP030778
*T.hainanicus* ^T^	Dai 17705	China	MZ484588	MZ437421
***T.indicus* ^T^**	**MUBL1083**	**India**	**OR272293**	**OR272338**
***T.indicus***	**MUBL1084**	**India**	**OR272294**	**OR272339**
*T.lineatus* ^T^	Dai 21196	Malaysia	MZ484594	MZ437426
*T.linteus*	JV 0904/64	USA	JQ860322	JX467701
*T.linteus*	JV 0904/140	USA	JQ860323	KP030780
*T.minor* ^T^	Dai 21139	China	MZ484592	MZ437424
*T.minus*	Dai 18487A	China	MZ484590	MZ437422
	Dai 21183	China	MZ484593	MZ437425
*T.nullisetus* ^T^	VXLF616	Brazil	MN795129	MN812261
*T.nullisetus*	VRTO195	Brazil	MN795118	MN812254
***T.pseudoindicus* ^T^**	**MUBL1087**	**India**	**OR272295**	**OR272340**
***T.pseudoindicus***	**MUBL1088**	**India**	**OR272296**	**OR272341**
*T.pseudolinteus*	JV0402/35-K	Venezuela	KC778781	MF772820
	JV 0312/22.10-J	Venezuela	KC778780	–
*T.ravidus* ^T^	Dai 18165	China	MZ484595	MZ437427
*T.rudis*	O 915614	Rwanda	KP030796	–
	O 915617	Tanzania	KP030797	MH101016
*T.sideroxylicola*	JV 1207/4.3-J	USA	KC778783	–
*T.sideroxylicola* ^T^	JV 0409/30-J	USA	KC778782	–
*T.stratificans* ^T^	SMDB 14732	Brazil	KM199689	–
*T.stratificans*	VRTO884	Brazil	MN795124	MN812266
*T.substratificans* ^T^	JV 1908/80	French Guiana	MZ484597	MZ437429
***T.tamilnaduensis* ^T^**	**MUBL1085**	**India**	**OR272297**	**OR272343**
***T.tamilnaduensis***	**MUBL1086**	**India**	–	**OR272344**
*T.tenuis* ^T^	Dai 19699	China	MZ484598	MZ437430
*T.tenuis*	Dai 19724	China	MZ484599	MZ437431
*T.texanus* ^T^	CBS 145357	USA	NR_168219	NG_068906
*T.texanus*	TX8	USA	MN108123	MN113949
*T.tropicalis*	UTHSC 02-617	USA	AY641432	–
	UAMH 10376	USA	AY599487	–

^T^ = Type material and “-” refers to the data unavailability. Sequences generated from the present study are indicated in bold.

### ﻿Genealogical concordance phylogenetic species recognition analysis

**Figure 1.**
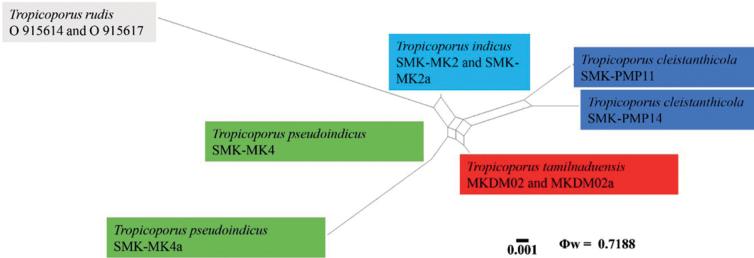
Split graphs show the results of the PHI test of the new species, *Tropicoporusindicus*, *T.tamilnaduensis*, *T.pseudoindicus*, *T.cleistanthicola* and their most closely-related species *T.rudis*, using LogDet transformation and split decomposition options. The PHI test result Φw ≤ 0.05 indicates that there is a significant recombination within the dataset.

Genealogical concordance phylogenetic species recognition analysis (GCPSR) by the pairwise homoplasy index (PHI) test was used to determine the recombination level within closely-related species (Bruen et al. 2006). The data were analysed by the software SplitsTree 4 (Bruen et al. 2006; Huson and Bryant 2006). The relationships between closely related taxa were visualised by constructing split graphs from concatenated datasets, using the LogDet transformation and splits decomposition options. If the PHI test value is (Φw) ≤ 0.05, it indicates significant recombination within the dataset. This is an important method to provide further evidence to justify a species. All results are shown in Fig. 1.

## ﻿Results

### ﻿Phylogenetic analyses

**Figure 2.**
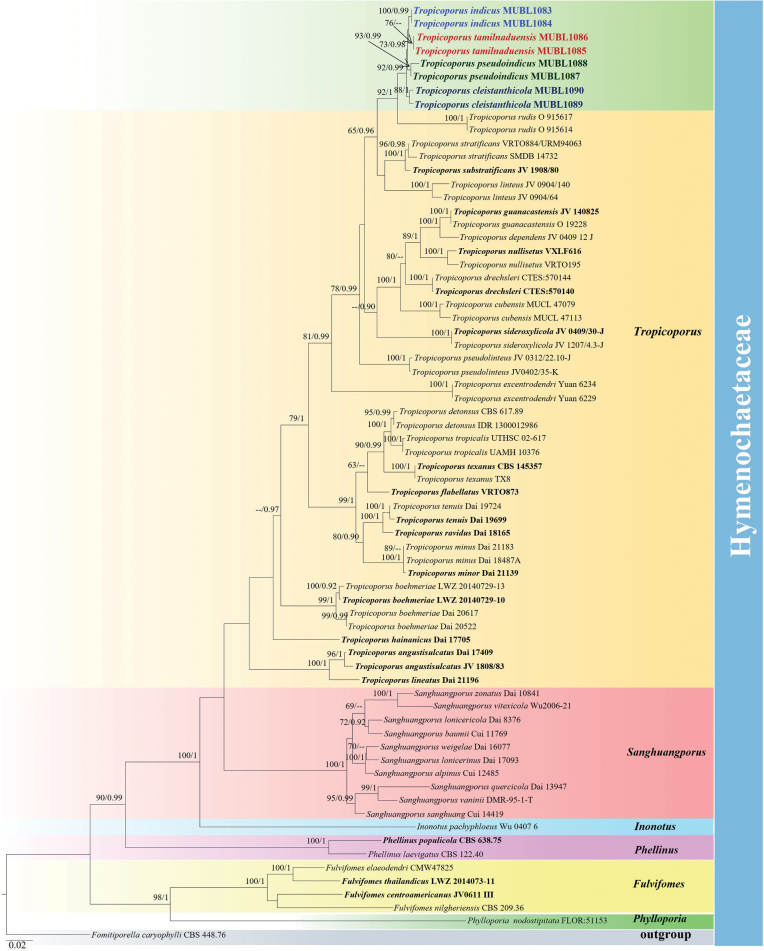
Molecular phylogeny of four new Indian *Tropicoporus* species and other hymenochaetoid species inferred from combined ITS and nLSU sequences. The topology is from the Bayesian analysis. Maximum Likelihood bootstrap values and Bayesian posterior probabilities, above 60% and 0.9, respectively, are labelled at the nodes. The newly-generated sequences are coloured and bold; the type specimens are in bold.

In total, eight new sequences of the ITS and seven new sequences of the nLSU regions were generated and submitted to GenBank (Table 1). Additionally, 62 taxa (52 nLSU and 62 ITS sequences) were retrieved from GenBank (Table 1). The combined nLSU and ITS dataset were aligned and the multiple sequence alignment consists of 1,820 characters (914 for nLSU and 902 for ITS) of which 1,017 were constant, 962 were variable and 570 (31%) were parsimony informative. The best-fit evolutionary model (GAMMA+P-Invar Model) was estimated by jModelTest 2.1.10 for the combined datasets. The Maximum Likelihood (ML) trees were constructed using raxmlGUI 2.0 with 1,000 rapid bootstrap inferences (BS). The Bayesian analysis was run for 2,000,000 generations and the average standard deviation reached 0.010166. The phylogenetic topology was selected from Bayesian analysis. The Maximum Likelihood bootstrap values ≥ 60% and the Bayesian posterior probabilities ≥ 0.90 are summarised in Fig. 2.

### ﻿Taxonomical descriptions of the four novel species of *Tropicoporus*

#### ﻿ Tropicoporus cleistanthicola

Taxon classificationFungiHymenochaetalesHymenochaetaceae

S. Gunaseelan & M. Kaliyaperumal sp. nov.

C47090F9-D091-510F-B1A1-E1866E10E9F9

 849484

Figs 3 , 4

##### Etymology.

The specific epithet *cleistanthicola* (Lat.) refers to the host *Cleistanthuscollinus*.

##### Diagnosis.

*Tropicoporuscleistanthicola* is characterised by perennial, effused-reflexed to pileate, applanate to triquetrous basidiome with narrowly zonate, glabrous, meagrely warted pilear surface, acute margin, homogenous context, mono-dimitic hyphal system, presence of cystidioles and subglobose to broadly ellipsoid basidiospores measuring 4.7–5.4 × 4.2–4.9 μm.

**Figure 3.**
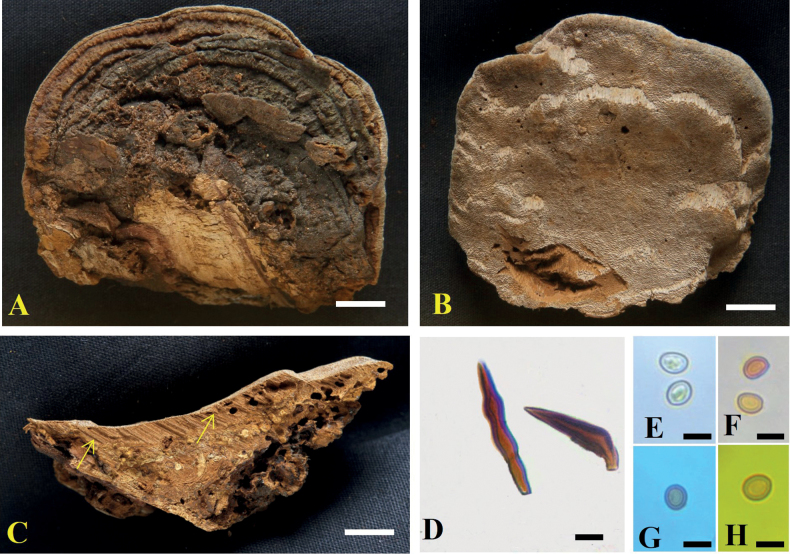
*Tropicoporuscleistanthicola* (MUBL1089 holotype) **A** basidiomata (Holotype) **B** pore surface **C** cross-section of basidiome (arrows indicating stratified tube layers) **D** hymenial setae **E–H** basidiospores: **E** basidiospores in water **F** basidiospores in KOH**G** basidiospore in cotton blue **H** basidiospore in Melzer’s reagent. Scale bars: 1 cm (**A–C**); 5 µm (**D–H**).

##### Type.

India, Tamil Nadu, Thiruvannamalai District, Jawadhu Hills, 12°54'24"N, 78°87'75"E; 15 Nov 2019; Sugantha Gunaseelan; on a living angiosperm tree (*Cleistanthuscollinus*); SMK-PMP11 (MUBL1089; Holotype); GenBank: OR272292 (ITS); OR272337 (nLSU).

**Figure 4.**
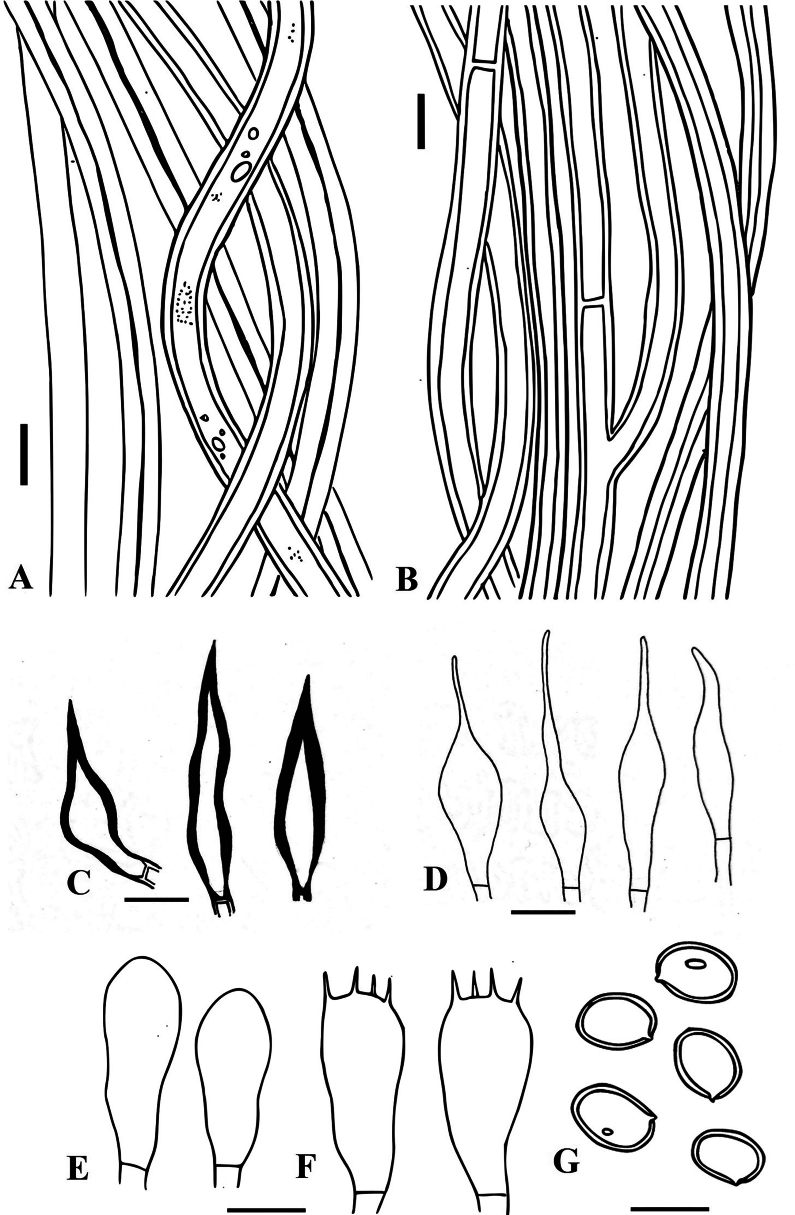
*Tropicoporuscleistanthicola* (MUBL1089 holotype) **A** contextual hyphae **B** tramal hyphae **C** hymenial setae **D** cystidioles **E** basidioles **F** basidia **G** basidiospores. Scale bars: 5 µm.

##### Description.

***Basidiome*** perennial, pileate, solitary, hard corky and without distinctive odour or taste when fresh, woody hard and light in weight when dry. ***Pilei*** effused-reflexed to pileate, dimidiate, triquetrous in section, projecting up to 4 cm, 6.5 cm wide and 3 cm thick at the base; Pileal surface narrowly zonate, glabrous, meagrely warted near attachment, yellowish-brown (5E6; 5E8) to dark brown (6F5), turning dark brown (7F4) to greyish-brown (6F3). ***Margin*** acute, 1 mm thick, light brown (6D5). ***Pore surface*** brown (6E6) to dark brown (6F7); sterile margin up to 2 mm wide, light brown (6D5); pores circular, 5–7 per mm. ***Context*** homogenous, up to 1.5 cm thick, brown (6E8). ***Tubes*** up to 0.5 cm long, tube layers distinctly stratified, each stratum up to 2 mm, brown (6E7).

##### Hyphal structures.

Hyphal system monomitic in the context and dimitic in the trama, tissue darkening with KOH without hyphal swelling.

##### Context.

Generative hyphae, thin to thick-walled, hyaline to golden yellow, simple septate, rarely branched, 2–5 μm diam.

##### Trama.

Generative hyphae, dominant, thin to thick-walled, hyaline to pale yellow, septate, occasionally branched, 2–4 μm diam. Skeletal hyphae thick-walled with narrow to wide lumen, yellowish-brown, aseptate, unbranched, 2–3.5 μm diam.

##### Hymenium.

Hymenial setae dark brown, thick-walled, ventricose to subulate with sharp to blunt tips, 5–32 × 4–5.5 μm. Cystidia absent. Cystidioles hyaline, thin-walled, ventricose to fusoid with elongated tapering apical portion, 7–45 × 2–5 μm. Basidia clavate to broadly clavate, 7–15 × 2.7–6.2 μm, with four sterigmata and a simple septum at the base. Basidioles clavate, 5–13 × 3.5–6 μm. Basidiospores broadly ellipsoid to subglobose, pale yellow in water, turning golden yellow to brown in KOH, thick-walled, smooth, CB ¯, IKI ¯, (4.7–) 4.9–5.2 (–5.4) × (4.2–) 4.5–4.7 (–4.9) μm (n = 50/2), Q = 1.1 (Q range 1.05–1.2).

##### Habitat and distribution.

Basidiomes were found on living trees of *Cleistanthuscollinus* (Phyllanthaceae), distributed in Jawadhu Hills, Thiruvannamalai District, Tamil Nadu, India.

##### Additional material examined.

India, Tamil Nadu, Thiruvannamalai District, Jawadhu Hills; 12°51'20"N, 78°73'71"E; 15 Nov 2019; Sugantha Gunaseelan; on a living angiosperm tree (*Cleistanthuscollinus*); SMK-PMP14 (MUBL1090, Paratype); GenBank: OR272291 (ITS); OR272336 (nLSU).

##### Notes.

The present phylogenetic study indicated that *T.cleistanthicola* is sister to *T.rudis* with significant support (92% ML/0.9 BPP). However, *T.rudis* has applanate basidiomes with fulvous, velvety, concentrically zonate, matted, rimose pilear surface, whereas *T.cleistanthicola* has triquetrous basidiome and glabrous pilear surface with infrequent warts without cracks. *Tropicoporuscleistanthicola* and *T.rudis* are comparable only in mono-dimitic hyphal system and *T.rudis* lacks cystidioles and has larger basidiospores (4.9−6 × 4−4.8 μm) (Wu et al. 2022). Despite sharing pileate basidiomes, mono-dimitic hyphal system and presence of cystidioles in *T.linteus*, *T.cleistanthicola* differs by having effused-reflexed to pileate, narrowly zonate, meagrely warted pilear surface (Tian et al. 2013; Wu et al. 2022). *Tropicoporuscleistanthicola* resembles *T.angustisulcatus*, *T.dependens*, *T.excentrodendri*, *T.substratificans* and *T.lineatus* by sharing pileate, triquetrous basidiomes with concentrically zonation and presence of cystidioles, but *T.cleistanthicola* differs by having a mono-dimitic hyphal system and spore size (4.7–5.4 × 4.2–4.9 μm) (Zhou et al. 2015; Wu et al. 2022). *Tropicoporuscleistanthicola* and *T.drechsleri* are similar in having pileate basidiomes and a mono-dimitic hyphal system with the presence of cystidioles, but *T.cleistanthicola* differs by having smaller pores (5–7 pores/mm) and larger basidiospores (Salvador-Montoya et al. 2018). Except for sharing a mono-dimitic hyphal system and indistinctly stratified tube layers, *T.cleistanthicola* differs from *T.flabellatus* and *T.guanacastensis* in pores (size and shape), basidiospore shape and absence of cystidioles (Zhou et al. 2015; Lima et al. 2022).

#### ﻿ Tropicoporus indicus

Taxon classificationFungiHymenochaetalesHymenochaetaceae

S. Gunaseelan & M. Kaliyaperumal sp. nov.

62082AE9-516E-542F-B4B6-1BFE5B345743

 849482

Figs 5 , 6

##### Etymology.

The species epithet “*indicus*” (Lat.): referring to the species being collected from India.

##### Diagnosis.

*Tropicoporusindicus* is characterised by applanate to meagrely triquetrous basidiome with concentrically zonate, sulcate, glabrous, deeply cracked to rimose pilear surface, homogenous context, acute margin, mono-dimitic hyphal system, presence of cystidioles, subglobose to broadly ellipsoid basidiospores measuring 5–6 × 4.2–4.9 μm.

**Figure 5.**
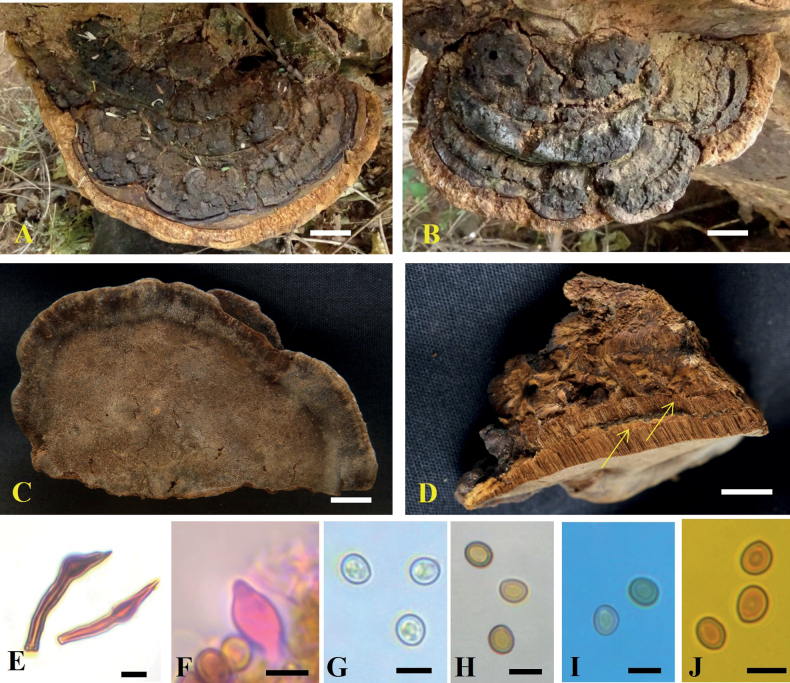
*Tropicoporusindicus* (MUBL1083 holotype) **A** basidiomata (Holotype) **B** basidiome (SMK-MK2a- Isotype) **C** pore surface **D** cross-section of basidiome (arrows indicating stratified tube layers) **E** hymenial setae **F** cystidioles **G–J** basidiospores: **G** basidiospores in water **H** basidiospores in KOH**I** basidiospores in cotton blue **J** basidiospores in Melzer’s reagent. Scale bars: 1 cm (**A–D**); 5 µm (**E–J**).

##### Type.

India, Tamil Nadu, Kallakurichi District, Kalvarayan Hills; 11°91'30"N, 78°57'86"E; 29 Sep 2022; Sugantha Gunaseelan; on living angiosperm tree of *Albiziaamara*; SMK- MK2 (MUBL1083, Holotype); GenBank: OR272293 (ITS); OR272338 (nLSU).

**Figure 6.**
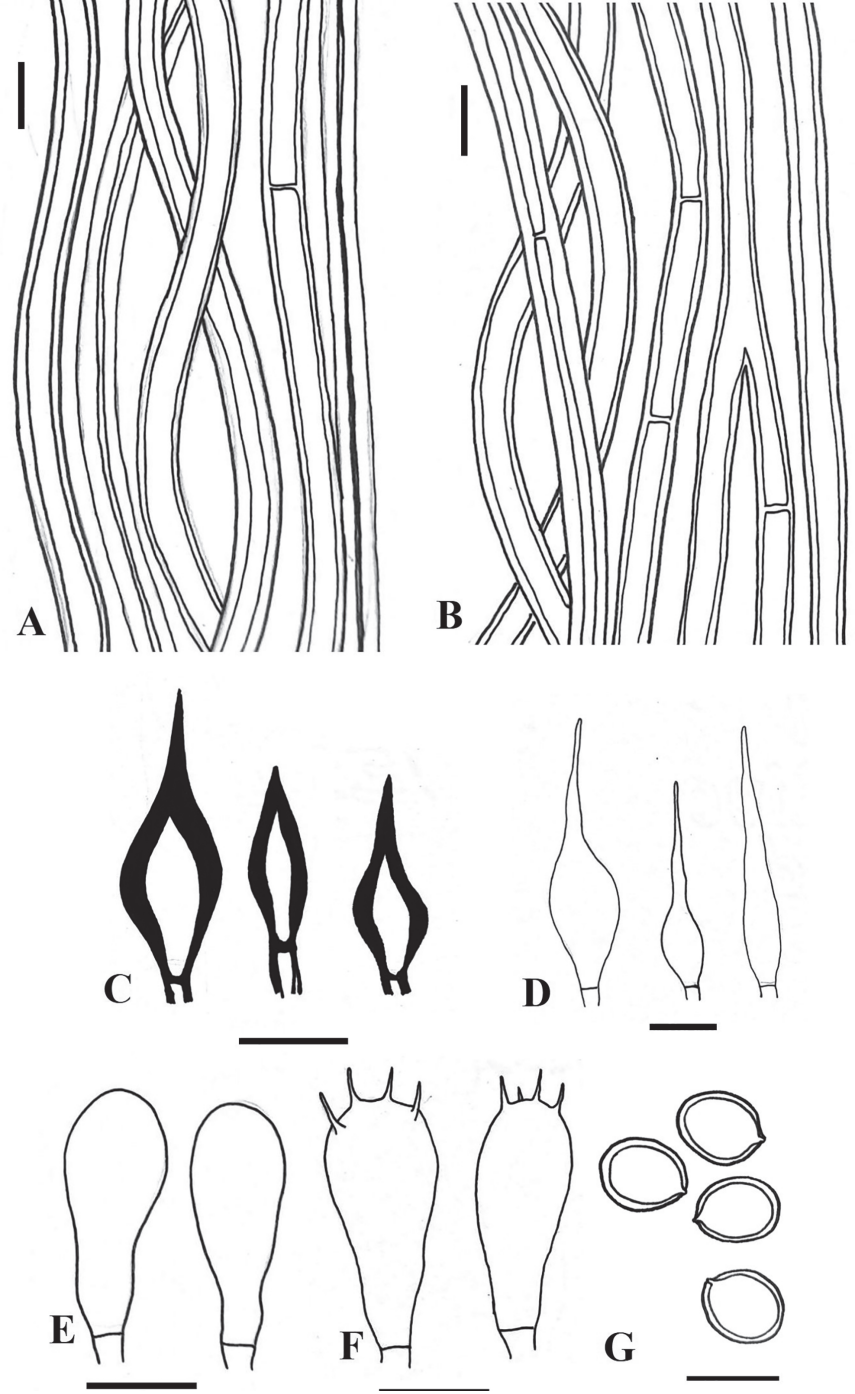
*Tropicoporusindicus* (MUBL1083 holotype) **A** contextual hyphae **B** tramal hyphae **C** hymenial setae **D** cystidioles **E** basidioles **F** basidia **G** basidiospores. Scale bars: 5 µm.

##### Description.

***Basidiome*** perennial, pileate, woody and without distinctive odour or taste when fresh, hard when dry. ***Pilei*** applanate to meagrely triquetrous, projecting up to 5.5 cm, 7 cm wide and 4 cm thick at the base; pileal surface concentrically zonate, sulcate, glabrous, deeply cracked to rimose near attachment, dark brown (7E4), greyish-brown (7F3). ***Margin*** velutinate, 2 mm thick, acute, brown (6E7). ***Pore surface*** light brown (5D5) to yellowish-brown (5E7); pores circular, 4–6 per mm. ***Context*** homogenous, up to 0.8 cm thick, brown (6E8) to dark brown (6F8). ***Tubes*** woody hard, up to 2.5 cm long, brown (6E7); tube layers stratified, each stratum up to 0.5 cm long.

##### Hyphal structures.

Hyphal system monomitic in the context and dimitic in the trama, tissue darkening with KOH without swelling

##### Context.

Generative hyphae, thin to thick-walled, hyaline to golden yellow, simple septate, rarely branched, 2–5 μm diam.

##### Trama.

Generative hyphae dominant, thin to thick-walled, hyaline to pale yellow, septate, occasionally branched, 2–4.5 μm diam. Skeletal hyphae thick-walled with narrow to wide lumen, yellowish-brown, aseptate, unbranched, 2–4 μm diam.

##### Hymenium.

Hymenial setae dark brown, thick-walled, ventricose to subulate with sharp to blunt tips, 7–28 × 3–5 μm. Cystidia absent. Cystidioles hyaline, thin-walled, ventricose to fusoid with elongated tapering apical portion, 5–21 × 3–5 μm. Basidia clavate to broadly clavate, 7–17 × 3–6 μm, with four sterigmata and a simple septum at the base. Basidioles clavate, 5–14 × 3–5 μm. Basidiospores broadly ellipsoid to subglobose, pale yellow in water, turning golden yellow to brown in KOH, thick-walled, smooth, CB ¯, IKI ¯, (5–) 5.3–5.8 (– 6) × (4.2–) 4.7–4.9 μm (n = 50/2), Q = 1.16 (Q range 1.05–1.3).

##### Habitat and distribution.

Basidiomes were found on living trees of Fabaceae members (*Albiziaamara* and *Prosopiscineraria*), distributed in Kalvarayan Hills, Kallakurichi District, Tamil Nadu, India.

##### Additional material examined.

India, Tamil Nadu, Kallakurichi District, Kalvarayan Hills; 11°90'39"N, 78°55'69"E; on a living angiosperm tree (*Prosopiscineraria*); 29 Sep 2022; Kezhocuyi Kezo; SMK-MK2a (MUBL1084, Paratype); GenBank: OR272294 (ITS); OR272339 (nLSU).

##### Notes.

Phylogenetically, *Tropicoporusindicus* was recovered in the *T.linteus* clade. *Tropicoporusindicus* is similar to *T.linteus* by sharing, pileate, dimidiate basidiomes, concentrically sulcate pilear surface, zonate context, smaller pores (5–7/mm), a mono-dimitic hyphal system and presence of cystidioles. While varying in the nature of cracks, *T.linteus* has more or less cracked basidiomes, *T.indicus* has irregular deep cracks in basidiomes, with larger basidiospores (*T.linteus* 4.8−5.7 × 3.8–4.8 μm and *T.indicus* 5–6 × 4.2–4.9 μm) (Tian et al. 2013; Wu et al. 2022). *Tropicoporusindicus* and *T.rudis* share a mono-dimitic hyphal system, but *T.indicus* differs from *T.rudis* in having zonate, sulcate, deeply cracked to rimose basidiomes and larger pores (4–6/mm). In these regards, *T.rudis* is characterised by fulvous, velvety, concentrically zonate, matted, rimose basidiome and smaller pores (6–7/mm) (Wu et al. 2022). *Tropicoporusindicus* differs from *T.angustisulcatus*, *T.excentrodendri*, *T.lineatus* and *T.substratificans* in having mono-dimitic hyphal system and a cracked basidiome (Zhou et al. 2015; Wu et al. 2022). *Tropicoporusindicus* and *T.flabellatus* are similar in having a mono-dimitic hyphal system, but differ significantly by having concentrical zones, sulcate, glabrous, deeply-cracked to rimose pilear surface with larger pores (4–6/mm) and broadly ellipsoid to subglobose spores (5–6 × 4.2–4.9 μm). *Tropicoporusflabellatus*, in contrast, has a velutinate pilear surface, uncracked basidiomes with smaller pores (7–9/mm) and smaller basidiospores (4.5–5 × 3.5–4 μm) (Lima et al. 2022). *Tropicoporusindicus* and *T.guanacastensis* are similar in having sulcate, cracked basidiome, stratified tube, mono-dimitic hyphal system and ventricose setae. However, the former differs in larger pores (4–6/mm) and larger basidiospores (*T.indicus* 5–6 × 4.2–4.9 μm vs. *T.guanacastensis* 4.1–5 × 3.1–4 μm) (Zhou et al. 2015). *Tropicoporusindicus* and *T.drechsleri* share concentrically sulcate deeply-cracked pilei with mono-dimitic hyphal system, larger pores (< 6/mm) and presence of cystidioles, but the South American species differs in basidiospore size (4–5.5 × 3–4.5 μm) (Salvador-Montoya et al. 2018).

#### ﻿ Tropicoporus pseudoindicus

Taxon classificationFungiHymenochaetalesHymenochaetaceae

S. Gunaseelan & M. Kaliyaperumal sp. nov.

EB804D33-3563-5FC4-B633-236EAF3383A6

 849483

Figs 7 , 8

##### Etymology.

The species *pseudoindicus* signifies the close morphological and phylogenetic relationships with the species *Tropicoporusindicus*.

##### Diagnosis.

*Tropicoporuspseudoindicus* is characterised by applanate to meagrely ungulate to triquetrous basidiome with broadly zonate, distinctly cracked by radial fissures, sulcate pilear surface, duplex context with black line, acute to obtuse margin, pores 6–8/mm, mono-dimitic hyphal system, presence of cystidioles, subglobose to broadly ellipsoid basidiospores measuring 4–5.2 × 3.7–4.7 μm.

**Figure 7.**
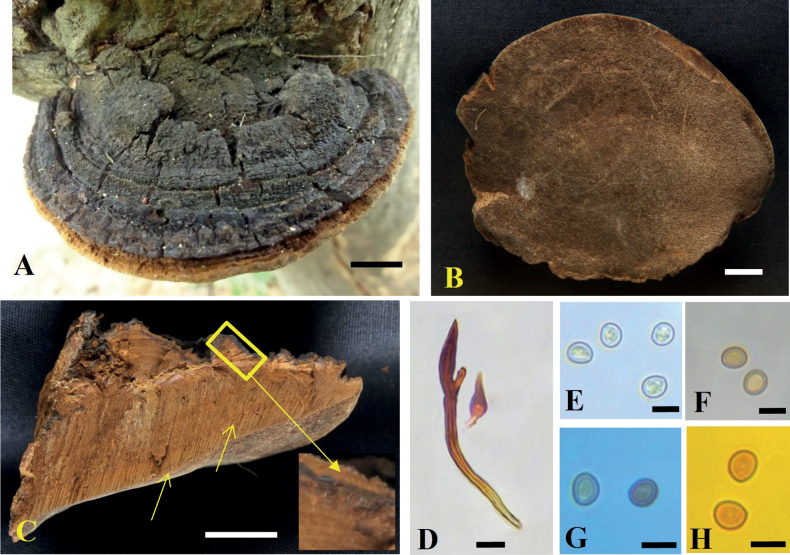
*Tropicoporuspseudoindicus* (MUBL1085 holotype) **A** basidiomata (Holotype) **B** pore surface **C** cross-section of basidiome (arrows indicating stratified tube layers and duplex context with black line) **D** hymenial setae **E–H** basidiospores: **E** basidiospores in water **F** basidiospores in KOH**G** basidiospores in cotton blue **H** basidiospores in Melzer’s reagent. Scale bars: 1 cm (**A–C**); 5 µm (**D–H**).

##### Type.

India, Tamil Nadu, Kallakurichi District, Kalvarayan Hills; 11°86'98"N, 78°55'68"E; 29 Sep. 2022; Sugantha Gunaseelan; on a living angiosperm tree (*Albiziaamara*); SMK-MK4 (MUBL1087, Holotype); GenBank: OR272295 (ITS); OR272340 (nLSU).

**Figure 8.**
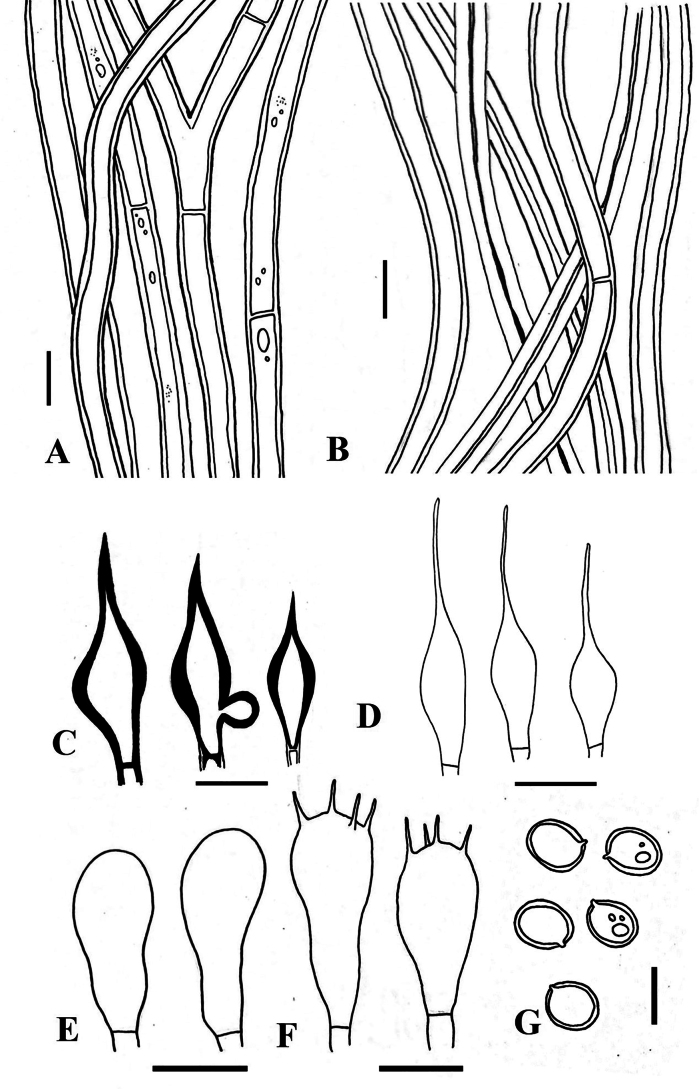
*Tropicoporuspseudoindicus* (MUBL1085 holotype) **A** contextual hyphae **B** tramal hyphae **C** hymenial setae **D** cystidioles **E** basidioles **F** basidia **G** basidiospores. Scale bars: 5 µm.

##### Description.

***Basidiome*** perennial, pileate, woody and without distinctive odour or taste when fresh, hard and light in weight when dry. ***Pilei*** applanate, meagrely ungulate to triquetrous, dimidiate, projecting up to 5 cm, 8 cm wide and 3.5 cm thick at base; pileal surface broadly zonate, distinctly cracked by radial fissures, sulcate, brown (6E8), dark brown (7F4) to greyish-brown (6F3). ***Margin*** acute to obtuse, up to 3 mm thick, light brown (6D5). ***Pore surface*** brown (6E6) to dark brown (7E6); sterile margin brown (6E6), up to 2 mm wide; pores circular, 6–8 per mm. ***Context*** duplex with black line, woody hard, up to 1.2 cm thick, several black lines present along context, brown (6E7) to dark brown (7F6). ***Tubes*** up to 1.5 cm long, annual layers distinct, each stratum up to 0.3 cm, brown (7E8) to dark brown (6F8).

##### Hyphal structures.

Hyphal system monomitic in the context and dimitic in the trama, tissue darkening with KOH without swelling.

##### Context.

Generative hyphae, thin to thick-walled, hyaline to golden yellow, simple septate, rarely branched, 2–5 μm diam.

##### Trama.

Generative hyphae, dominant, thin to thick-walled, hyaline to pale yellow, septate, occasionally branched, 2–4 μm diam. Skeletal hyphae, thick-walled with narrow to wide lumen, yellowish-brown, aseptate, unbranched, 2–3.5 μm diam.

##### Hymenium.

Hymenial setae dark brown, thick-walled, ventricose to subulate with sharp to blunt tips, rarely with lateral appendage, 5–18 × 3–5.5 μm. Cystidia absent. Cystidioles hyaline, thin-walled, ventricose to fusoid with elongated tapering apical portion, 7–52 × 2.5–5.2 μm. Basidia clavate to broadly clavate, 7–15 × 2.7–6.2 μm, with four sterigmata and a simple septum at the base. Basidioles clavate, 5–13 × 3.5–6 μm. Basidiospores broadly ellipsoid to subglobose, pale yellow in water, turning golden yellow to brown in KOH, thick-walled, smooth, CB ¯, IKI ¯, (4–) 4.2–5 (–5.2) × (3.7–) 4–4.5 (–4.7) μm (n = 50/2) and Q = 1.14 (Q range 1.05–1.25).

##### Habitat and distribution.

Basidiomes were found on living trees of Fabaceae members (*Albiziaamara* and *Peltophorumpterocarpum*), distributed in Kalvarayan Hills, Kallakurichi District, Tamil Nadu, India.

##### Additional material examined.

India, Tamil Nadu, Kallakurichi District, Kalvarayan Hills; 11°87'33"N, 78°42'78"E; 29 Sep 2022; Kezhocuyi Kezo; on a living angiosperm tree (*Peltophorumpterocarpum*); SMK-MK4a (MUBL1088, Paratype); GenBank: OR272296 (ITS); OR272341 (nLSU).

##### Notes.

*Tropicoporuspseudoindicus* and *T.drechsleri*, share similar characteristics, such as applanate basidiomes with mono-dimitic hyphal system and the presence of cystidioles; however, *T.pseudoindicus* differs in having smaller pores (6–8/mm) and larger basidiospores (*T.pseudoindicus* 4–5.2 × 3.7–4.7 μm vs. *T.drechsleri* 4–5.5 × 3–4.5 μm) (Salvador-Montoya et al. 2018). *Tropicoporuspseudoindicus* resembles *T.rudis* in having a mono-dimitic hyphal system, presence of cystidioles, and basidiospore shape, but differs from *T.rudis* in having distinctly cracked, fissured to sulcate pilei, duplex context and smaller basidiospores (4–5.2 × 3.7–4.7 μm) (Vlasák et al. 2013). *Tropicoporuspseudoindicus* differs from *T.guanacastensis* in having cystidioles and subglobose to broadly ellipsoidal spores (*T.pseudoindicus* 4–5.2 × 3.7–4.7 μm vs. *T.guanacastensis* 4.1–5.0 × 3.1–4.0 μm) (Tian et al. 2013). *Tropicoporuspseudoindicus* and *T.linteus* share similar pileate basidiomes with sulcate pilear surface, smaller pores (5–7/mm), mono-dimitic hyphal system and presence of cystidioles. However, the former differs in basidiospore size (*T.linteus* 4.8−5.7 × 3.8–4.8 μm vs. *T.pseudoindicus* 4–5.2 × 3.7–4.7 μm) (Tian et al. 2013; Wu et al. 2022). *Tropicoporuspseudoindicus* and *T.flabellatus* are similar in their mono-dimitic hyphal system, but differ significantly in pilear characteristics, absence of cystidioles and size and shape of basidiospores (*T.flabellatus* 4.5–5 × 3.5–4 μm vs. *T.pseudoindicus* (4–) 4.2–5 (–5.2) × (3.7–) 4–4.5 (–4.7) μm) (Lima et al. 2022). *Tropicoporuspseudoindicus* differs from some other reported *Tropicoporus* species (namely *T.angustisulcatus*, *T.excentrodendri*, *T.lineatus* and *T.substratificans*) in having concentrically zonate, glabrous, distinctly cracked pileal surface and a mono-dimitic hyphal system (Zhou et al. 2015; Wu et al. 2022).

#### ﻿ Tropicoporus tamilnaduensis

Taxon classificationFungiHymenochaetalesHymenochaetaceae

M. Kaliyaperumal & S. Gunaseelan sp. nov.

3B319787-3A32-5422-BA78-63D0C49F04A6

 849481

Figs 9 , 10

##### Etymology.

The species epithet *tamilnaduensis* refers to the locality of the type specimen (Tamil Nadu).

##### Diagnosis.

*Tropicoporustamilnaduensis* is characterised by applanate to meagrely ungulate basidiome with glabrous, broadly zonate, sulcate and deeply irregularly cracked pilear surface, homogenous context, obtuse margin, pores 4–5/mm, mono-dimitic hyphal system, presence of cystidioles, subglobose to broadly ellipsoid basidiospores measuring 4.5–5.7 × 3.5–4.7 μm.

**Figure 9.**
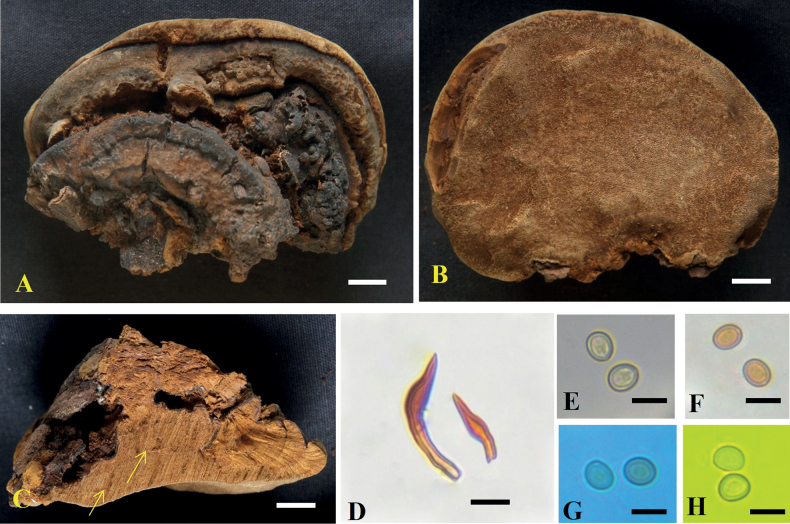
*Tropicoporustamilnaduensis* (MUBL1085 holotype) **A** basidiomata (Holotype) **B** pore surface **C** cross-section of basidiome (arrows indicating stratified tube layers) **D** hymenial setae **E–H** basidiospores: **E** basidiospore in water **F** basidiospores in KOH**G** basidiospores in cotton blue **H** basidiospores in Melzer’s reagent. Scale bars: 1 cm (**A–C**); 5 µm (**D–H**).

##### Type.

India, Tamil Nadu, Cuddalore District, Thaiyalkunampattinam, Kanni Tamil Nadu; 11°59'18"N, 79°60'17"E; 31 Dec 2022; Malarvizhi Kaliyaperumal; on a living angiosperm tree (*Madhucalongifolia*); MKDM02 (MUBL1085, holotype); GenBank: OR272297 (ITS); OR272343 (nLSU).

**Figure 10.**
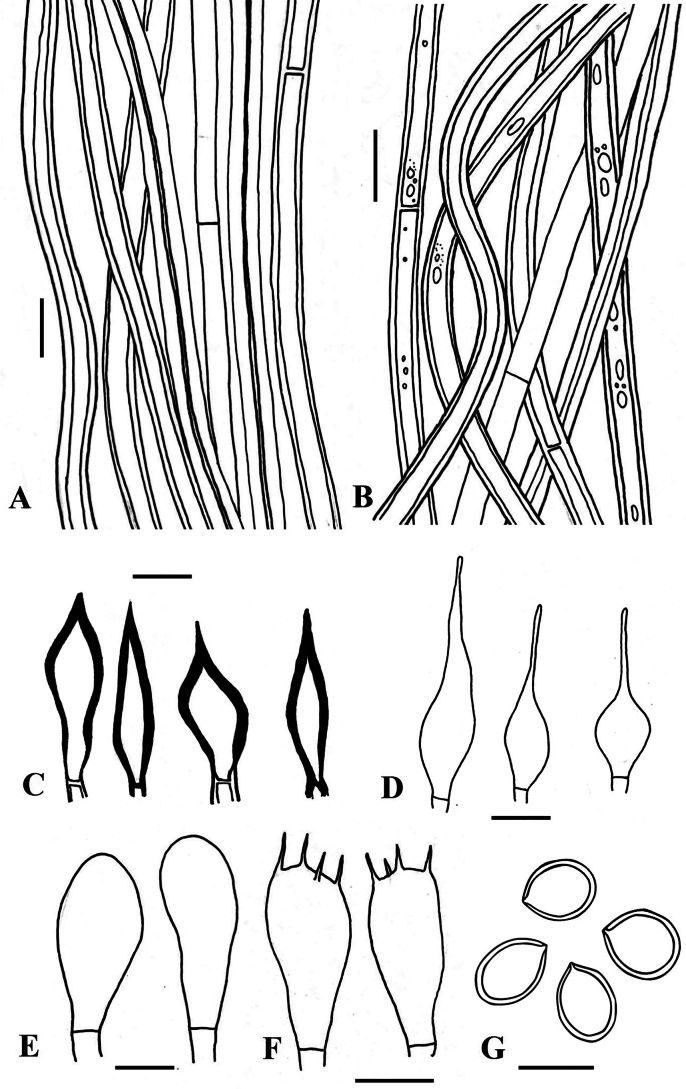
*Tropicoporustamilnaduensis* (MUBL1085 holotype) **A** tramal hyphae **B** contextual hyphae **C** hymenial setae **D** cystidioles **E** basidioles **F** basidia **G** Basidiospores. Scale bars: 5 µm.

##### Description.

***Basidiome*** perennial, pileate, without distinctive odour or taste when fresh, woody hard and light in weight when dry. ***Pilei*** applanate to meagrely ungulate, projecting up to 5 cm, 8 cm wide and 4 cm thick at base; pileal surface glabrous, broadly zonate, sulcate, deeply irregularly cracked near attachment, brown (6E7), yellowish-brown (5F4) to golden brown (7F7) turning greyish-brown (5F3). ***Margin*** obtuse, 4 mm thick, light brown (6D5). ***Pore surface*** brown (6E6), sterile margin yellowish-brown (5E6), up to 2 mm wide; pores circular, 4–5 per mm; dissepiments thick, entire. ***Context*** homogenous, zonate, brown (6D7) to dark brown (6F8), woody hard, up to 2 cm thick. ***Tubes*** brown (6E6), up to 2 cm long, annual layers distinct, each stratum up to 0.3 cm long.

##### Hyphal structures.

Hyphal system monomitic in the context and dimitic in the trama, tissue darkening with KOH without hyphal swelling.

##### Context.

Generative hyphae, thin to thick-walled, hyaline to golden yellow, simple septate, rarely branched, 2–5 μm diam.

##### Trama.

Generative hyphae, dominant, thin to thick-walled, hyaline to pale yellow, septate, occasionally branched, 2–4 μm diam. Skeletal hyphae, thick-walled with narrow to wide lumen, yellowish-brown, aseptate, unbranched, 2–3.5 μm diam.

##### Hymenium.

Hymenial setae dark brown, thick-walled, ventricose to subulate with sharp to blunt tips, 6–19 × 3.8–5 μm. Cystidia absent. Cystidioles hyaline, thin walled, ventricose to fusoid with elongated tapering apical portion, 10–45 × 2–5 μm. Basidia clavate to broadly clavate, 7–15 × 2.7–6.2 μm, with four sterigmata and a simple septum at the base. Basidioles clavate, 5–13 × 3.5–6 μm. Basidiospores broadly ellipsoid to subglobose, pale yellow in water, turning golden yellow to brown in KOH, thick-walled, smooth, CB ¯, IKI ¯, (4.5–) 4.7–5.5 (–5.7) × (3.5–) 3.7–4.5 (–4.7) μm (n = 50/2), Q = 1.13 (Q range 1.05–1.25).

##### Habitat and distribution.

Basidiomes are found on living trees of *Madhucalongifolia* and *Prosopiscineraria*, distributed in Cuddalore District, Tamil Nadu, India.

##### Additional material examined.

India, Tamil Nadu, Cuddalore District, Thaiyalkunampattinam, Kanni Tamil Nadu; 11°50'14"N, 79°54'14"E; 31 Dec 2022; Malarvizhi Kaliyaperumal; on a living angiosperm tree (*Prosopiscineraria*); MKDM02a (MUBL1086, Paratype); GenBank: OR272344 (nLSU).

##### Notes.

*Tropicoporustamilnaduensis* and *T.linteus* are similar in having pileate sulcate basidiomes, zonate context and a mono-dimitic hyphal system with cystidioles. However, *T.tamilnaduensis* differs from *T.linteus* in deeply-cracked basidiomes and smaller basidiospores (Tian et al. 2013; Wu et al. 2022). *Tropicoporustamilnaduensis* and *T.rudis* share a homogenous context, a mono-dimitic hyphal system and subglobose to broadly ellipsoid basidiospores, but *T.tamilnaduensis* differs in having zonate, sulcate, deeply irregularlycracked basidiome and larger pores (4–5/mm) (Wu et al. 2022). *Tropicoporustamilnaduensis* differs from *T.angustisulcatus*, *T.lineatus* and *T.substratificans* in having sulcate and deeply cracked basidiomes and a mono-dimitic hyphal system, while *T.angustisulcatus*, *T.lineatus* and *T.substratificans* have basidiomes with velutinate to glabrous, uncracked pilear surface and a dimitic hyphal system (Zhou et al. 2015; Wu et al. 2022). *Tropicoporustamilnaduensis* resembles *T.excentrodendri*, *T.dependens* and *T.sideroxylicola* by sharing concentrically zonate, sulcate, cracked to rimose pilear surface; however, the former differs by having a mono-dimitic hyphal system (Zhou et al. 2015; Salvador-Montoya et al. 2018). *Tropicoporustamilnaduensis* and *T.guanacastensis* are similar in having a mono-dimitic hyphal system; however, *T.tamilnaduensis* has ungulate, sulcate, deeply irregularly cracked basidiomes with larger pores (4–7/mm) and broadly ellipsoid to subglobose spores (4.5–5.7 × 3.5–4.7 μm) (Zhou et al. 2015). Although sharing a mono-dimitic hyphal system, *T.tamilnaduensis* differs from *T.flabellatus* by having sulcate, deeply irregularly cracked basidiomes, cystidioles and larger pores (4–7/mm) with homogenous context (Lima et al. 2022). *Tropicoporustamilnaduensis* varies from *T.drechsleri* by having sulcate, deeply irregularly cracked basidiomes and broadly ellipsoid to ellipsoid basidiospores (4.5–5.7 × 3.5–4.7 μm) (Salvador-Montoya et al. 2018).

## ﻿Discussion

Recently, the *Inonotuslinteus* complex has gained attention because of its medicinal values and as an emerging potential pathogen in plants (Dai et al. 2009; Dai 2010), humans (Sutton et al. 2005; Haidar et al. 2017; Gupta et al. 2022) and dogs (Hevia et al. 2019). Zhou et al. (2015) segregated the *I.linteus* complex into two new genera viz. *Sanghuangporus* Sheng H. Wu, L.W. Zhou & Y.C. Dai and *Tropicoporus* L.W. Zhou, Y.C. Dai & Shen. H. Wu. Since then, many new species/combinations from tropical countries, especially from China followed by the Americas were introduced (Salvador-Montoya et al. 2018; Brown et al. 2019; Wu et al. 2022). *Tropicoporus* is characterised by its annual to perennial, resupinate, effused-reflexed to pileate basidiomes with mono-dimitic, dimitic hyphal system, ellipsoid to subglobose basidiospores. To date, twenty-three legitimate species are accepted under *Tropicoporus*, of which eleven were from tropical American countries, seven were from East Asian countries and one each from Africa, Costa Rica, Cuba and French Guiana.

The Bayesian phylogram illustrated in the present study is consistent with the previous studies (Coelho et al. 2016; Salvador-Montoya et al. 2018; Brown et al. 2019; Lima et al. 2022). The four new *Tropicoporus* species from Tamil Nadu, India, fit well within the *Tropicoporus* clade but formed a unique, distinct lineage that was the sister clade to *T.rudis* (earlier treated as *Xanthochrousrudis*). The *T.rudis* clade consists of strictly African collections (92% BS, 1.00 BPP) in the phylogeny (Fig. 1). This clade, in turn, forms the sister clade to a clade composed of *T.stratificans*, *T.substratificans* and *T.linteus* with 62% BS, 0.96 BPP.

The Eastern Ghats has discontinuous mountain ranges with hills ranging from 1,100 to 1,600 m with luxuriant vegetation of tropical evergreen to deciduous, thorn forest or scrub jungle that harbours diverse groups of wood rot fungi. This is the first report of the genus *Tropicoporus* from the Eastern Ghats of Tamil Nadu with three novel species, viz. *T.cleistanthicola*, *T.indicus* and *T.pseudoindicus*.

*Tropicoporuscleistanthicola*, *T.tamilnaduensis*, *T.indicus* and *T.pseudoindicus* are characterised by their perennial, pileate basidiomes with mono-dimitic hyphal system, presence of cystidioles and hymenial setae, smooth, thick-walled, coloured and inamyloid basidiospores (Table 2). However, there is significant variation in their basidiome characteristics, pore (shape and size) and basidiospore (shape and size). *Tropicoporuscleistanthicola* differs from the other three species in having effused-reflexed to pileate, sulcate, and narrowly zonate basidiome with infrequent warts. *Tropicoporustamilnaduensis* differs in irregularly cracked basidiome with glabrous, sulcate and irregularly cracked, ungulate basidiome and smaller basidiospores (4.5–5.4 × 3.5–4.7 μm), while *T.indicus* has regularly cracked and concentrical zonate basidiome, glancing pore surface and larger basidiospores (5–6 × 4.2–4.9 μm). Morphologically, *T.pseudoindicus* is similar to *T.indicus* in sharing concentrically zonate, glabrous and rimose with maturity, mono-dimitic hyphal system and presence of cystidioles, while *T.pseudoindicus* differs by having sulcate deeply cracked, radially fissured basidiome, homogenous context and smaller basidiospores. Our Indian *Tropicoporus* species (*Tropicoporuscleistanthicola*, *T.tamilnaduensis*, *T.indicus* and *T.pseudoindicus*) could be easily distinguished by its pileate basidiomes and mono-dimitic hyphal system from the other *Tropicoporus* resupinate species (*T.boehmeriae*, *T.hainanicus*, *T.minus*, *T.ravidus*, *T.stratificans*, *T.tenuis* and *T.texanus*) (Wu et al. 2015; Coelho et al. 2016; Brown et al. 2019; Wu et al. 2022).

**Table 2.** Synoptic comparison of characteristics amongst species of the newly-reported *Tropicoporus* from India.

Species	Basidiomata	Context	Margin	Pores /mm	Hyphal system	Setae (in μm)	Cystidioles (in μm)	Basidiospores (in μm)	Q value (Q range)
*T.cleistanthicola*	Effused-reflexed to pileate, applanate to triquetrous basidiome with narrowly zonate, glabrous, meagrely warted pilear surface	Homogenous	Acute	5–7	Mono-Di	5–32 × 4–5.5	7–45 × 2–5	(4.7–) 4.9–5.2 (–5.4) × (4.2–) 4.5–4.7 (–4.9)	1.1 (1.05–1.2)
*T.indicus*	Applanate to meagrely triquetrous basidiome with concentrically zonate, sulcate, glabrous, deeply cracked to rimose pilear surface	Homogenous	Acute	4–6	Mono-Di	7–28 × 3–5	5–21 × 3–5	(5–) 5.3–5.8 (– 6) × (4.2–) 4.7–4.9	1.16 (1.05–1.3)
*T.pseudoindicus*	Applanate, meagrely ungulate to triquetrous basidiome with broadly zonate, distinctly cracked by radial fissures, sulcate pilear surface	Duplex with blackline	Acute to obtuse	6–8	Mono-Di	5–18 × 3–5.5	7–52 × 2.5–5.2	(4–) 4.2–5 (–5.2) × (3.7–) 4–4.5 (–4.7)	1.14 (1.05–1.25)
*T.tamilnaduensis*	Applanate to meagrely ungulate basidiome with glabrous, broadly zonate, sulcate and deeply irregularly cracked pilear surface	Homogenous	Obtuse	4–5	Mono-Di	6–19 × 3.8–5	10–45 × 2–5	(4.5–) 4.7–5.4 (–5.7) × (3.5–) 3.7–4.5 (–4.7)	1.13 (1.05–1.25)

### ﻿Key to species of *Tropicoporus* in the Afro-Asian region

**Table:** 

1	Basidiocarps resupinate to effused-reflexed	**2**
–	Basidiocarps distinctly pileate	**7**
2	Basidiocarps annual to biennial	**3**
–	Basidiocarps perennial	**6**
3	Basidiospores cyanophilic	***T.tenuis***
–	Basidiospores acyanophilic	**4**
4	Basidiocarp resupinate to effused reflexed, pileal surface tomentose to hispid basidiospores > 3 μm in length	***T.excentrodendri***
–	Basidiocarp resupinate, basidiospores < 3 μm in length	**5**
5	Dissepiments lacerate, context layer present between tube layers	***T.hainanicus***
–	Dissepiments entire, context layer absent between tub layers	***T.boehmeriae***
6	Basidiocarp resupinate, cystidioles present, pores 10–12/mm	***T.minus***
–	Basidiocarp cushion-shaped, cystidioles absent, pores 8–10/mm	***T.ravidus***
7	Hyphal system strictly dimitic	***T.lineatus***
–	Hyphal system mono-dimitic, dimitic in trama	**8**
8	Context homogenous	**9**
–	Context duplex with black line	***T.pseudoindicus***
9	Effused reflexed to pileate, uncracked basidiome	***T.cleistanthicola***
–	Applanate to ungulate or triquetrous, cracked pilear surface	**10**
10	Pores > 6/mm, cyanophilic basidiospores	***T.rudis***
–	Pores < 6/mm, acyanophilic basidiospores	**11**
11	Applanate to triquetrous basidiome with acute velutinate margin, regularly cracked pilear surface	***T.indicus***
–	Applanate to meagrely ungulate basidiome with obtuse margin and deeply irregularly cracked pilear surface	***T.tamilnaduensis***

## Supplementary Material

XML Treatment for Tropicoporus cleistanthicola

XML Treatment for Tropicoporus indicus

XML Treatment for Tropicoporus pseudoindicus

XML Treatment for Tropicoporus tamilnaduensis

## ﻿Additional information

### Author ORCIDs

Sugantha Gunaseelan https://orcid.org/0000-0001-7089-2292

Kezhocuyi Kezo https://orcid.org/0000-0002-3723-0462

Samantha C. Karunarathna https://orcid.org/0000-0001-7080-0781

Erfu Yang https://orcid.org/0000-0003-2385-6402

Changlin Zhao https://orcid.org/0000-0002-1395-591X

Abdallah M. Elgorban https://orcid.org/0000-0003-3664-7853

Saowaluck Tibpromma https://orcid.org/0000-0002-4706-6547

Malarvizhi Kaliyaperumal https://orcid.org/0000-0002-1218-3778

### Data availability

All holotype and paratype collections of the new species are deposited at Madras University Botany Laboratory (MUBL), Centre for Advanced Studies in Botany, University of Madras, Chennai-600 025, Tamil Nadu, India. The sequences generated during this study are deposited in NCBI GenBank. The ITS and nLSU alignment is deposited in TreeBase.

## Supplementary materials

Supplementary material 1 Pairwise distance matrix, based on nucleotide sequences of four new *Tropicoporus* spp. and its related species Data type xls Explanation note Pairwise distance matrix, based on nucleotide sequences of four new *Tropicoporus* spp. and its related species (Pairwise distances calculations were accomplished using MEGA X v.10.0.2. Distances and standard errors are respectively displayed in the lower-left matrix and the upper-right matrix). https://binary.pensoft.net/file/979932This dataset is made available under the Open Database License (http://opendatacommons.org/licenses/odbl/1.0/). The Open Database License (ODbL) is a license agreement intended to allow users to freely share, modify, and use this Dataset while maintaining this same freedom for others, provided that the original source and author(s) are credited.Sugantha Gunaseelan, Kezhocuyi Kezo, Samantha C. Karunarathna, Erfu Yang, Changlin Zhao, Abdallah M. Elgorban, Saowaluck Tibpromma, Malarvizhi Kaliyaperumal

Supplementary material 2 Molecular Phylogeny of four new Indian *Tropicoporus* species inferred from ITS sequences Data type jpg Explanation note The topology is from Bayesian analysis. Bootstrap values and Bayesian posterior probabilities, equal to or above 60% and 0.90, respectively, are labelled at the nodes. The newly-generated sequences are coloured and bold, and the type specimens are in bold. https://binary.pensoft.net/file/979933This dataset is made available under the Open Database License (http://opendatacommons.org/licenses/odbl/1.0/). The Open Database License (ODbL) is a license agreement intended to allow users to freely share, modify, and use this Dataset while maintaining this same freedom for others, provided that the original source and author(s) are credited.Sugantha Gunaseelan, Kezhocuyi Kezo, Samantha C. Karunarathna, Erfu Yang, Changlin Zhao, Abdallah M. Elgorban, Saowaluck Tibpromma, Malarvizhi Kaliyaperumal

Supplementary material 3 Molecular Phylogeny of four new Indian *Tropicoporus* species inferred from nLSU sequences Data type jpg Explanation note The topology is from Bayesian analysis. Bootstrap values and Bayesian posterior probabilities, equal to or above 60% and 0.90, respectively, are labelled at the nodes. The newly-generated sequences are coloured and bold and the type specimens are in bold. https://binary.pensoft.net/file/979934This dataset is made available under the Open Database License (http://opendatacommons.org/licenses/odbl/1.0/). The Open Database License (ODbL) is a license agreement intended to allow users to freely share, modify, and use this Dataset while maintaining this same freedom for others, provided that the original source and author(s) are credited.Sugantha Gunaseelan, Kezhocuyi Kezo, Samantha C. Karunarathna, Erfu Yang, Changlin Zhao, Abdallah M. Elgorban, Saowaluck Tibpromma, Malarvizhi Kaliyaperumal
