# Supplementary figures and images for: ﻿New species of Tropicoporus (Basidiomycota, Hymenochaetales, Hymenochaetaceae) from India, with a key to Afro-Asian Tropicoporus species
Source: MycoKeys. 2024 Feb 5;102:29–54. doi: 10.3897/mycokeys.102.117067 (PMC10862346; doi:10.3897/mycokeys.102.117067)

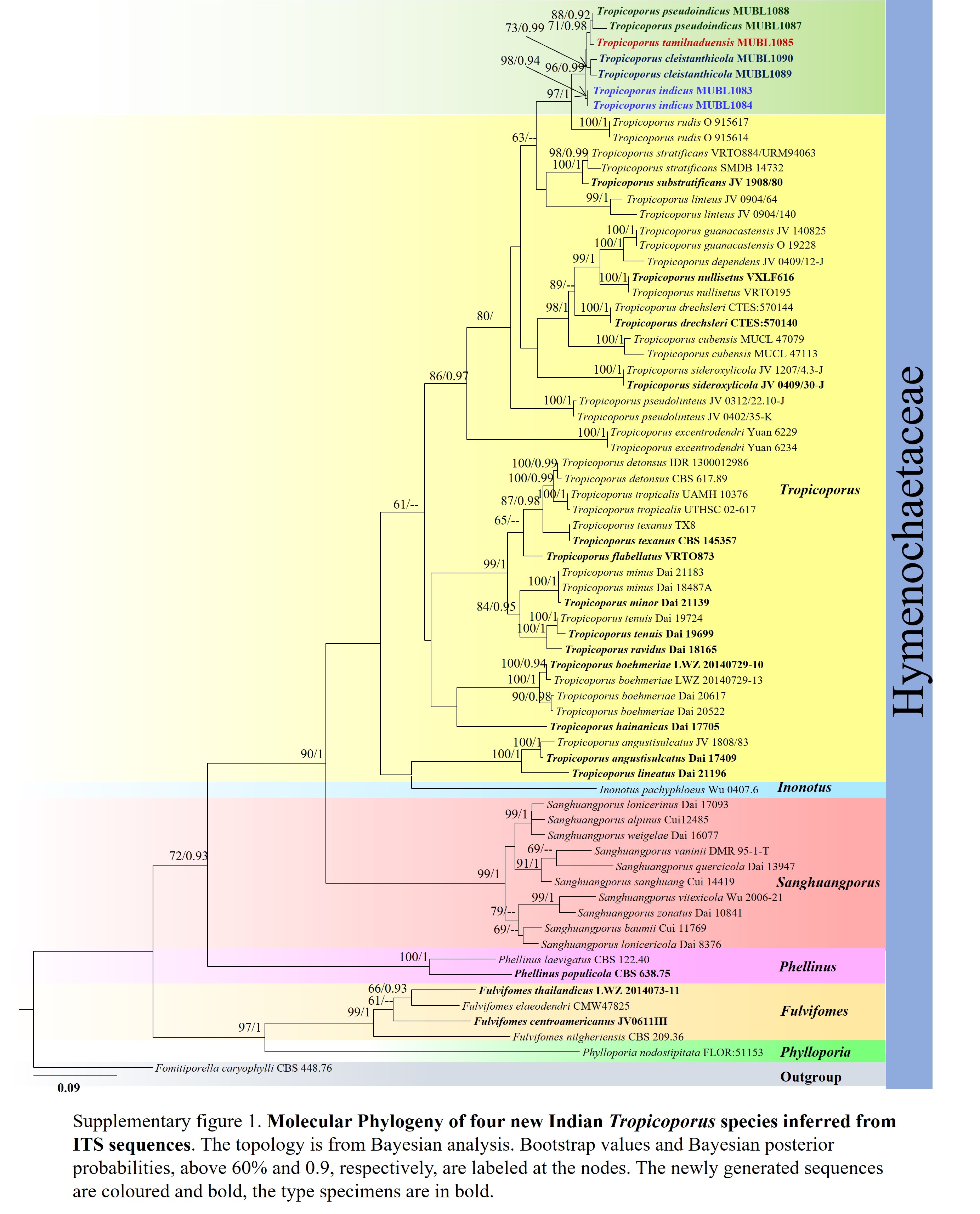

Supplement: Supplementary material 2 — Molecular Phylogeny of four new Indian Tropicoporus species inferred from ITS sequences [file mycokeys-102-029-s002.jpg]

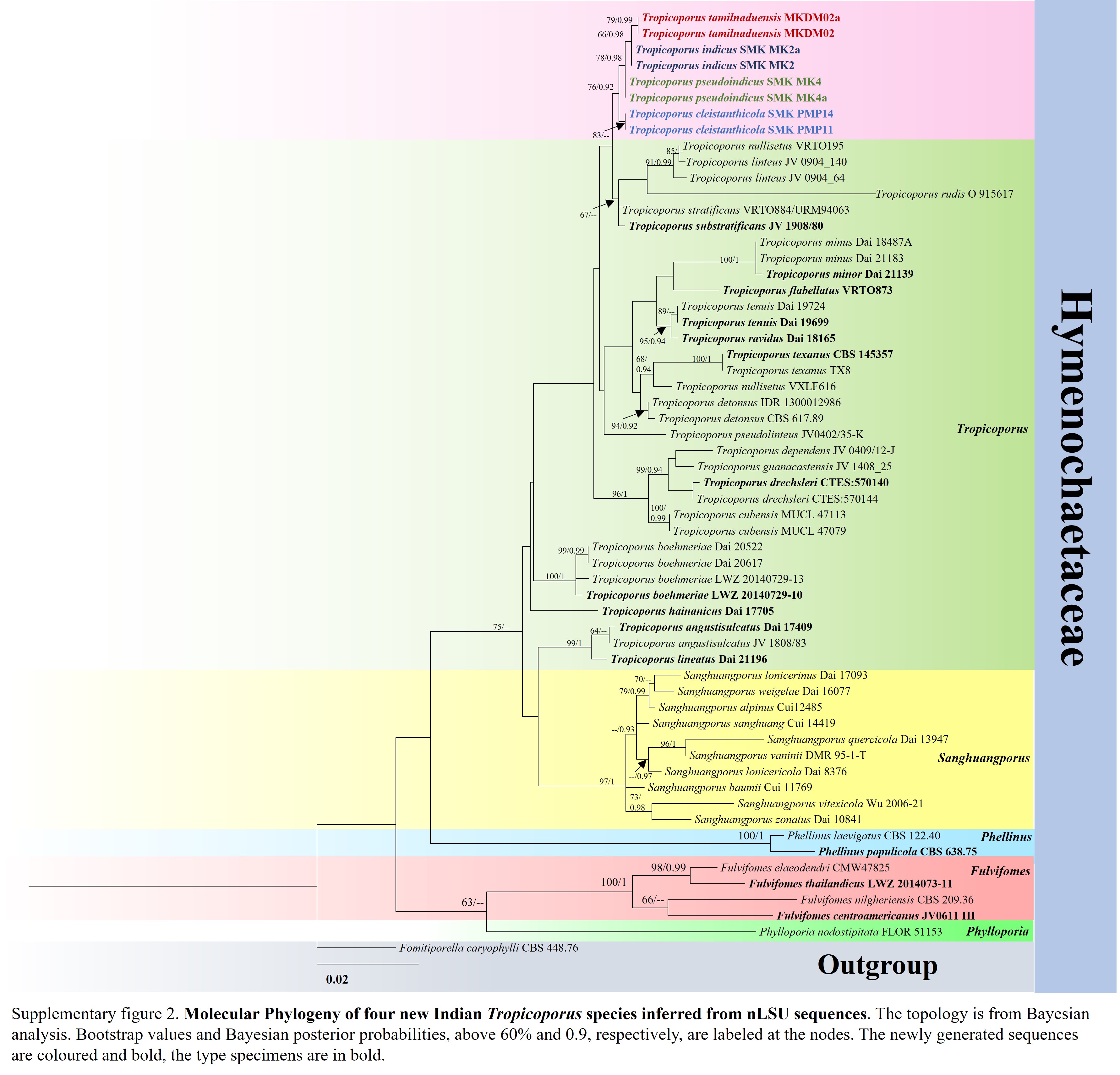

Supplement: Supplementary material 3 — Molecular Phylogeny of four new Indian Tropicoporus species inferred from nLSU sequences [file mycokeys-102-029-s003.jpg]
